# Development and characterization of cell models harbouring mtDNA deletions for *in vitro* study of Pearson syndrome

**DOI:** 10.1242/dmm.049083

**Published:** 2022-03-01

**Authors:** Carmen Hernández-Ainsa, Ester López-Gallardo, María Concepción García-Jiménez, Francisco José Climent-Alcalá, Carmen Rodríguez-Vigil, Marta García Fernández de Villalta, Rafael Artuch, Julio Montoya, Eduardo Ruiz-Pesini, Sonia Emperador

**Affiliations:** 1 Departamento de Bioquímica, Biología Molecular y Celular, Universidad de Zaragoza, 50013 Zaragoza, Spain; 2 Instituto de Investigación Sanitaria de Aragón (IIS-Aragón), 50009 Zaragoza, Spain; 3 Centro de Investigaciones Biomédicas en Red de Enfermedades Raras (CIBERER), 28029 Madrid, Spain; 4 Servicio de Pediatría. Hospital Universitario Miguel Servet, 50009 Zaragoza, Spain; 5 Unidad de Patología Compleja, Servicio de Pediatría. Hospital Universitario La Paz, 28046 Madrid, Spain; 6 Clinical Biochemistry, Genetics, Pediatric Neurology and Neonatalogy Departments, Institut de Recerca Sant Joan de Déu, 08950 Barcelona, Spain

**Keywords:** Pearson syndrome, Mitochondrial DNA, mtDNA deletion, Mitochondrial disease, Cybrid, iPSCs

## Abstract

Pearson syndrome is a rare multisystem disease caused by single large-scale mitochondrial DNA deletions (SLSMDs). The syndrome presents early in infancy and is mainly characterised by refractory sideroblastic anaemia. Prognosis is poor and treatment is supportive, thus the development of new models for the study of Pearson syndrome and new therapy strategies is essential. In this work, we report three different cell models carrying an SLMSD: fibroblasts, transmitochondrial cybrids and induced pluripotent stem cells (iPSCs). All studied models exhibited an aberrant mitochondrial ultrastructure and defective oxidative phosphorylation system function, showing a decrease in different parameters, such as mitochondrial ATP, respiratory complex IV activity and quantity or oxygen consumption. Despite this, iPSCs harbouring ‘common deletion’ were able to differentiate into three germ layers. Additionally, cybrid clones only showed mitochondrial dysfunction when heteroplasmy level reached 70%. Some differences observed among models may depend on their metabolic profile; therefore, we consider that these three models are useful for the *in vitro* study of Pearson syndrome, as well as for testing new specific therapies.

This article has an associated First Person interview with the first author of the paper.

## Abstract (precis)

**Summary:** Comparison of three different cell models from Pearson syndrome patients harbouring mitochondrial DNA deletions, which showed a severe failure in OXPHOS function, thus confirming their usefulness for therapeutic research.

## INTRODUCTION

Pearson syndrome (OMIM, 557000) is a rare genetic disorder characterised by pancytopenia, with vacuolization of bone marrow precursors and exocrine pancreatic dysfunction in early infancy (Pearson et al., 1979). It is caused by single large-scale mitochondrial DNA deletions (SLSMDs), leading to severe defects in the mitochondrial respiratory chain (Rötig et al., 1995). Heteroplasmy, a situation referring to mutant and wild-type mitochondrial DNA (mtDNA) co-existence, is obligatory in this pathology. The severity of the disease and phenotypic heterogeneity is defined by the degree and distribution of heteroplasmy in affected tissues (Cherry et al., 2013). Several factors, such as mtDNA deletion size, deletion location and skeletal muscle mtDNA heteroplasmy, are prognostic of disease severity and evolution (Grady et al., 2014).

Pearson syndrome rarely occurs as a result of inherited mutations from a maternal heteroplasmic progenitor oocyte that may harbour a low proportion of deleted mtDNA (Shanske et al., 2002). As practically all cases are sporadic, there is no gene therapy to prevent the onset of the disease. Therefore, treatment is the only possible tool, being to date limited. Therapy is supportive and based on pancreatic extracts, fat-soluble vitamins (ADEK), transfusions and treatment of endocrinopathies, metabolic decompensations and other stress-triggering factors (Farruggia et al., 2018). Therefore, research in this field and, more specifically, the development of cellular and animal models for the study of the origin, development and treatment of this pathology is essential.

Few disease models carrying deleted mtDNA are currently available. Only an animal model developed for the study of mtDNA deletions is reported in the literature: the mito-miceΔ generated by Inoue et al., from which several publications have emerged (Inoue et al., 2000; Nakada and Hayashi, 2011; Katada et al., 2013). Regarding cell models, cybrids, generated by fusing platelets or enucleated fibroblasts with a cell line devoid of mtDNA (ρ° cells), have been the most used model (Hayashi et al., 1991; Porteous et al., 1998; Van Den Ouweland et al., 2000). Although some studies on fibroblasts and induced pluripotent stem cells (iPSCs) bearing mtDNA deletions have also been reported (Bourgeron et al., 1993; Van De Corput et al., 1997; Van Den Ouweland et al., 2000; Cherry et al., 2013; Russell et al., 2018; Peron et al., 2021), comparative analysis among different cell types with different deletions have not been previously described. In addition, studies in cell types that allow differentiation into specific tissues affected in Pearson syndrome patients are scarce.

In this work, we developed and characterised several cell models from two patients affected by Pearson syndrome: fibroblast, cybrids and iPSCs. Fibroblasts and iPSCs keep the nuclear and mitochondrial genetic background from patients, and iPSCs are able to differentiate into specific tissues. Cybrids present an aberrant genetic background but they allow the homogenisation of the nuclear genome to test the pathogenicity of mtDNA mutations. Our lines were carriers of two types of deletion: the most frequently reported mtDNA deletion, also called ‘common deletion’, that is found in patients with Kearns–Sayre, progressive external ophthalmoplegia (PEO) or Pearson syndrome (Holt et al., 1988; Larsson et al., 1990; McShane et al., 1991; Rötig et al., 1995); and a larger one that reaches 6514 base pairs (bp) and has not been described previously. Parameters, such as percentage of heteroplasmy, deletion size, mitochondrial biochemical involvement and mitochondrial morphology, were analysed and compared among different cell lineages. These experimental models reflect biochemical defects generated by mtDNA deletions; therefore, they are all useful for the *in vitro* study of Pearson syndrome. Our results show for the first time how the same deletion causes an oxidative phosphorylation (OXPHOS) defect in three cell models with different metabolic profiles, allowing their comparison, which makes them especially interesting for future studies on the development of new therapies for this disease.

## RESULTS

### Cases report

**Fig. 1.**
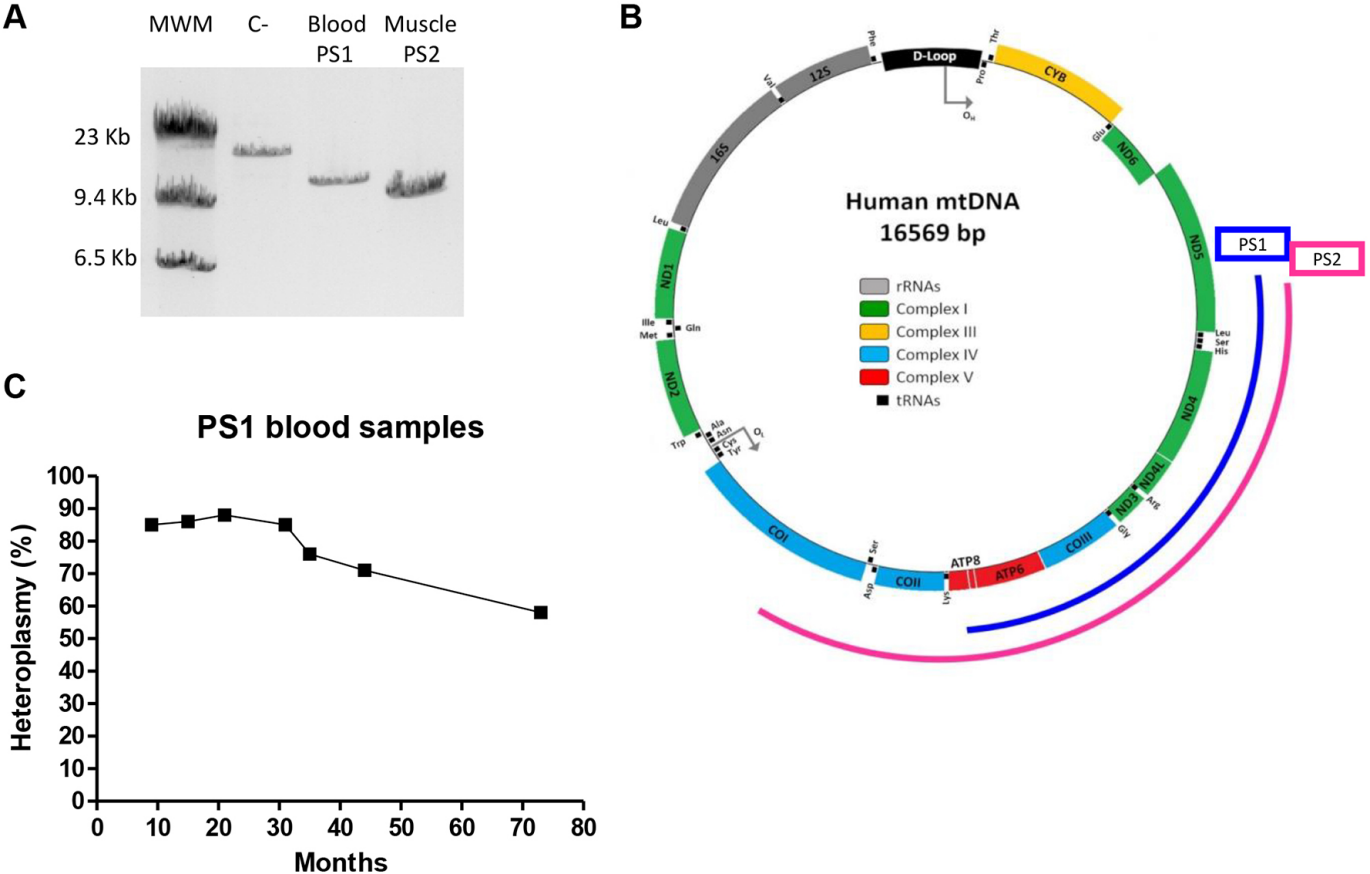
**Genetic characterization of mtDNA deletions in patients' samples.** (A) Gel showing long-range PCR results for PS1 blood and PS2 muscle samples. MWM, molecular weight marker; C-, negative control. (B) Schematic representation of the mitochondrial genome and the location of PS1 and PS2 deletions. (C) Graph showing the variation of heteroplasmy over time in different PS1 blood samples (measured by qPCR).

Patient 1 (PS1) is a 5-year-old male born at term by caesarean section from non-consanguineous and healthy Spanish parents. His older brother was healthy. Early psychomotor development was normal. At 3 months, he was diagnosed with anaemia and required blood transfusions from 8 months. Upon multiple hospitalizations, symptoms suggested a mitochondrial disease. We confirmed this clinical suspicion by detecting and mapping the mtDNA ‘common deletion’ in a blood sample, from nucleotide position 8469 to 13,447 (Fig. 1A,B). As expected, his mother was not carrying a mitochondrial deletion. Over 4 years, seven different blood analyses were performed. The deletion load remained at 85% until the patient was 30 months old. Surprisingly, a progressive reduction of heteroplasmy to 60% was detected in the following years as he became non-transfusion dependent (Fig. 1C). At the age of 2 years old, urine and oral mucous samples analyses revealed a 95% deletion.

Patient 2 (PS2) was a 5-year-old male born at term from non-consanguineous and healthy Spanish parents. An older sister was healthy. From the 30th week of pregnancy, a delayed intrauterine growth was detected. The delivery was induced at term and eutocic, but he was diagnosed with anaemia 48 h after birth. At 8 months, sideroblastic anaemia and other symptoms evoked Pearson syndrome, which was confirmed by the detection and mapping of a previously undescribed mtDNA deletion of 6514 bp, from nucleotide position 6897 to 13,411 (Fig. 1A,B). An imperfect directly repeated sequence of 6 or 7 bp was found flanking the mtDNA deletion. Exocrine pancreatic problems did not appear until he was 3 years old, accompanied by multiple endocrinopathies, atrioventricular conduction disorder, renal involvement and progressive neurological deterioration​. He died from a general dysfunction at 5 years of age. Several tissues were then analysed by Southern blotting and qPCR, and all tissues showed a high deletion rate, such as liver, which had 90% mutant mtDNA.

Ethical approval was obtained from the involved Institutional Review Boards. Written informed consent from the patients and the family members were also obtained.

### Genetic characterization of fibroblasts, cybrids and iPSCs harbouring single large-scale mtDNA deletions

**Fig. 2.**
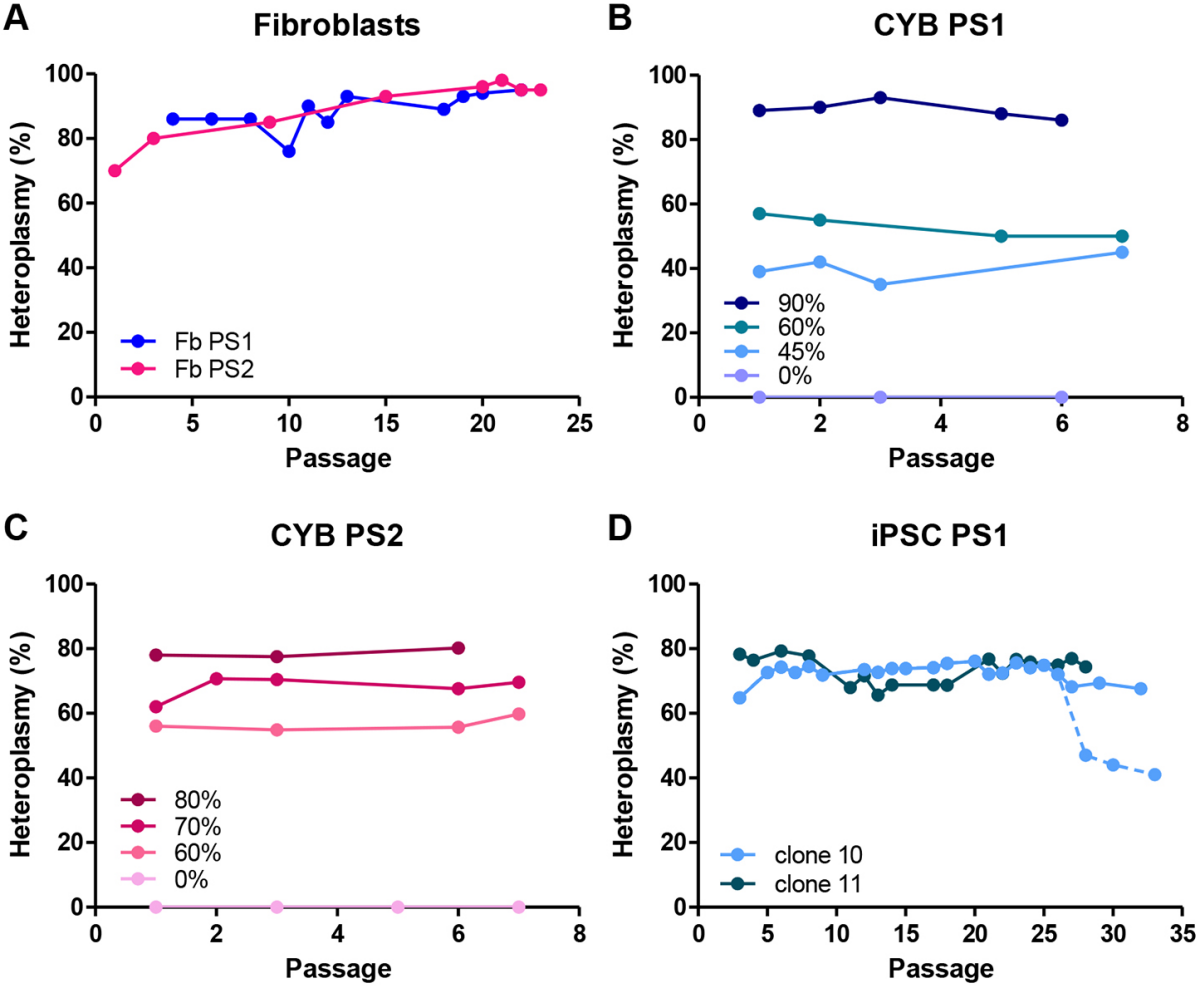
**Heteroplasmy levels of mtDNA deletions in the different cell models, as** **measured by qPCR.** (A) Fibroblasts PS1 and PS2. (B) Cybrid PS1. (C) Cybrid PS2. (D) iPSC PS1 clone 10 and 11. The dashed line shows the drop in the heteroplasmy level of iPSC PS1 clone 10. In B and C, the *x*-axes represents consecutive passages after mtDNA level recovery.

Several cell models were generated from Pearson syndrome patients: fibroblasts, cybrids and iPSCs. Nuclear and mitochondrial genomes were checked to confirm the identity and suitability of each line. Primary fibroblast culture from patients PS1 and PS2 was established in our laboratory. The deleted mtDNA load in the skin-derived fibroblasts was very high, reaching 95% for PS1 fibroblasts and 98% for PS2 fibroblasts along culture passes (Fig. 2A). Cybrid cell lines were generated to homogenise nuclear background. Clones with different and quite stable degrees of heteroplasmy were obtained for both lines [cybrid (CYB) PS1, 0%, 45%, 60% and 90%, and CYB PS2, 0%, 60%, 70%, 80%; Fig. 2B,C). iPSCs were generated by reprogramming the fibroblasts of patients (iPSC PS1 and iPSC PS2). Two clones with 70-75% of mitochondrial deletion, iPSC PS1 clone 10 and iPSC PS1 clone 11, were obtained from the ‘common deletion’-carrying patient (Fig. 2D). The study on the variation of the deletion load and mtDNA levels showed a stability over 20 passes. Deletion heteroplasmy was maintained at ∼75%, slightly lower than that present in the fibroblasts used for reprogramming. Some variations in heteroplasmy levels were observed during prolonged culture of these cells (Fig. 2D). Despite trying to generate an iPSC harbouring an mtDNA deletion from the fibroblasts of PS2 with high heteroplasmy levels, only a non-deletion bearing clone was obtained (iPSC PS2) after three independent reprogramming experiments.

Karyotype analysis of fibroblasts and iPSCs was carried out. Control fibroblasts and iPSCs, Pearson syndrome fibroblasts, iPSC PS1 clone 10 and iPSC PS2 were euploid (46,XY) but iPSC PS1 clone 11 showed a 45,X karyotype presenting a monosomy in sex chromosomes (Fig. S1). The nuclear background of osteosarcoma 143B had been checked previously (Gómez-Durán et al., 2010). We also confirmed, by DNA fingerprinting analysis, the genetic identity of cybrids and iPSCs derived from osteosarcoma 143B and the fibroblasts of patients, respectively (Table S1). mtDNA sequencing in all the lines confirmed the mitochondrial haplogroup of each control and patient, and the presence of the deletion in each case (Genbank MW927711 and MW927712 for controls, MW927713 and MW927714 for PS1 wild type and mutant, and MW927715 and MW927716 for PS2 wild type and mutant).

For each cell type, controls of the same lineage were generated and used in the different tests carried out. For fibroblasts, one control line belonging to the mitochondrial haplogroup U was used. Additional control cells of the same gender and similar age to both patients were not available. For cybrids, an isogenic control clone from each patient was generated and used (PS1 and PS2 patients were from haplogroup H and U, respectively). For iPSCs, two control clones derived from control fibroblasts from haplogroup H were used (clone V and clone G).

### Pearson syndrome iPSCs show differentiation capacity to the three germ layers

iPSCs can differentiate to specific tissues in which mitochondrial function can be studied. As manifestations of Pearson syndrome vary among different tissues, iPSCs offer a great number of possibilities to further bed in the biology of the disease.

Several reprogramming experiments and culture condition changes were necessary to obtain two common deletion-bearing iPSC clones from PS1 fibroblasts (Fig. 2D). However, clones without deletion were generated by the reprogramming of the 6514-bp deletion carrier line (PS2), despite performing the identical process.

**Fig. 3.**
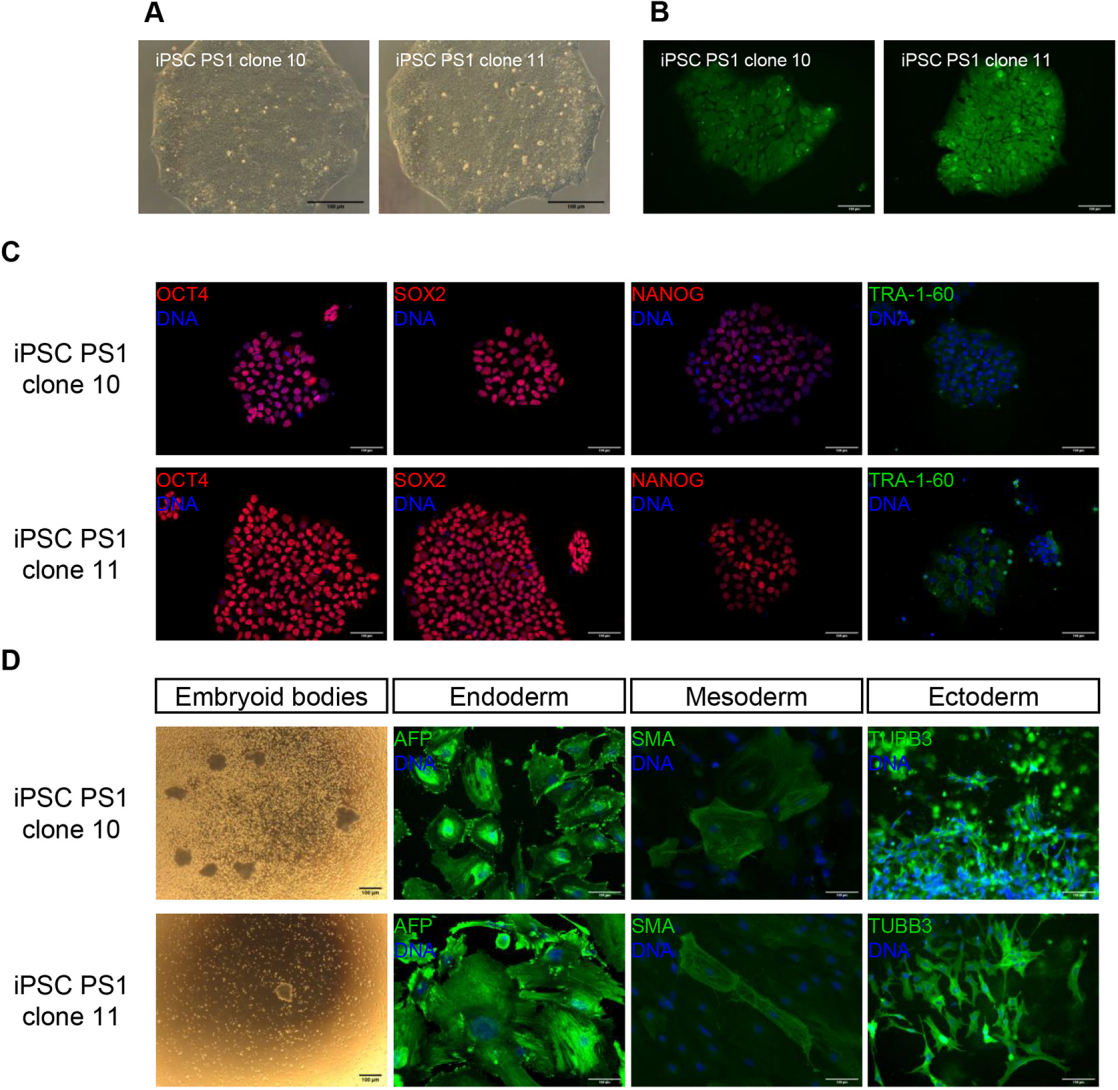
**Pluripotency characterisation of iPSC clones harbouring ‘common deletion’.** (A) Typical ESC-like colony morphology. (B) Positive alkaline phosphatase staining. (C) Immunofluorescence analysis of pluripotency-associated markers OCT4, SOX2, NANOG and TRA-1-60. (D) *In vitro* differentiation into embryoid bodies and all three germ layers showing positive staining for AFP in endoderm, SMA in mesoderm and TUBB3 in ectoderm. Scale bars: 100 µm.

The two mutant clones obtained (iPSC PS1 clone 10 and iPSC PS1 clone 11) behaved similarly. iPSC PS1 colonies presented a typical embryonic stem cell (ESC)-like morphology (Fig. 3A) and showed alkaline phosphatase activity (Fig. 3B), proposed as the most valid pluripotency marker in ESCs with correct morphology (Takahashi et al., 2007). Immunofluorescence analysis revealed the expression of transcription factors OCT4 (also known as POU5F1), SOX2, NANOG and surface marker TRA-1-60, which are characteristics of ESCs (Fig. 3C). Each line was able to generate embryoid bodies and showed the capacity to differentiate into the three embryonic germ layers (endoderm, mesoderm and ectoderm) detected by the immunofluorescence analysis of specific markers (Fig. 3D). Heteroplasmy levels of iPSC PS1 were maintained upon differentiation into the three germ layers (70-75%). Therefore, the presence of the deletion did not seem to impair the pluripotency of the defective line.

### mtDNA deletion causes severe effects on OXPHOS function in deletion carrier lines

To confirm that all our models are effective at revealing the mitochondrial defect caused by mtDNA deletions, several parameters were studied using high deletion level carrier lines (above 75%). Data from iPSC PS1 clone 11 are not shown because its behaviour was similar to clone 10.

**Fig. 4.**
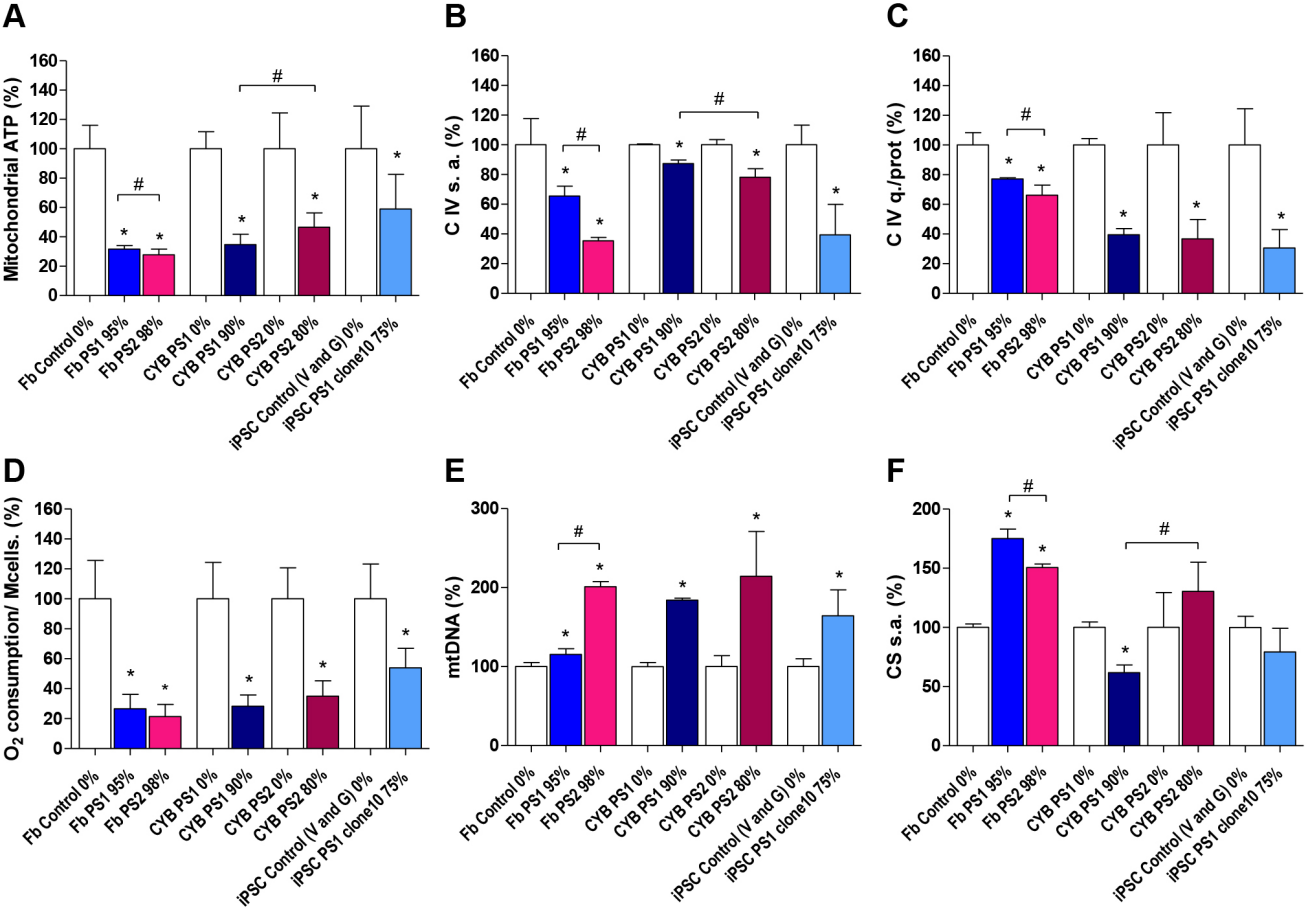
**OXPHOS function in the different cell models carrying mtDNA deletions.** (A) Mitochondrial ATP levels [fibroblast (Fb), *N*=12; CYB PS1, *N*=6; CYB PS2, *N*=11; iPSC, *N*=28]. (B) Respiratory CIV specific activity (s.a.) (*N*=3). (C) CIV quantity (q.) normalised for total protein (*N*=3). (D) Endogenous oxygen consumption normalised for millions of cells (Mcells) (*N*=3). (E) mtDNA copy number (*N*=3). (F) Citrate synthase (CS) specific activity (*N*=3). The heteroplasmy level of every line is indicated after the name. Bars indicate the mean value of each line relative to that of corresponding control cells in percentage and s.d. of independent experiments. Statistically significant differences were determined by a Mann–Whitney test (*P*<0.05) and are represented by * (versus control line) and # (versus Pearson syndrome lines).

Fibroblasts, cybrids and iPSCs harbouring mtDNA deletion presented a significant decrease in mitochondrial ATP levels and complex IV (CIV) activity and quantity compared to controls (Fig. 4A-C). We observed that PS2 fibroblasts and cybrids showed lower CIV activity than PS1, which could be caused by its larger deletion (Fig. 4B,C). However, the amount of mitochondrial ATP did not seem to be affected as much by the size of the deletion, as suggested by the contradictory results obtained between PS1 and PS2 fibroblasts and cybrids (Fig. 4A). Although the drop in mitochondrial ATP was more serious in the PS2 fibroblast, the fall in cybrid lines was more severe in the line with the common deletion that presented a slightly higher deletion load.

The baseline oxidative respiration rate was reduced in all cellular models carrying deletion (Fig. 4D). This decrease in the respiration rate was higher than 50% in fibroblasts and cybrids but close to 40% in iPSC lines, probably due to the lower mutation load in the pluripotent line or the different metabolic profile of each cell type.

mtDNA copy number was significantly increased in all mutant cells, most likely due to an ineffective compensatory strategy to increase the missing mitochondrial subunits and, thus, reduce negative effects of the deletion (Fig. 4E). Previous results suggested that mitochondrial biogenesis is also a compensatory strategy in mitochondrial dysfunction (Giordano et al., 2014). We observed that citrate synthase activity, used as a marker of mitochondrial mass, was significantly increased in Pearson syndrome fibroblasts, but not altered or even decreased in Pearson syndrome iPSCs and cybrids (Fig. 4F). Therefore, not all parameters linked to mitochondrial biogenesis are always co-presented with mtDNA deletions. Different tendencies observed among Pearson syndrome cell models demonstrated that a drop in OXPHOS parameters would not depend on mitochondrial mass, so citrate synthase activity was not used to normalise them, although it could limit the interpretation of results. Other authors also did not find differences in mitochondrial content in their studies performed in cybrids with mitochondrial deletion (Porteous et al., 1998) or fibroblasts carrying tRNA point mutations, even suggesting an increase in the degradation of mitochondria (James et al., 1996). Perhaps studying parameters such as mitophagy rate or mitochondrial turnover would also be interesting in our lines.

### Bioenergetic threshold level is above 60% in Pearson syndrome cybrids carrying different deletions

In mitochondrial diseases, cellular energy and OXPHOS activity decline, while the percentage of mutant mtDNA molecules increases. A minimum energy output is necessary for a cell or tissue to have normal function. When energy is below this bioenergetic threshold, disease appears. The threshold level of heteroplasmy may be different for distinct mtDNA mutations and even for the same mutation in different tissues (Wallace, 1999). Cybrid lines with different mitochondrial mutation load were studied to understand the level of mutation that the cell can tolerate to have normal function. mtDNA deletion percentages of 45, 60 and 90% were studied in PS1 cybrid (Fig. 2B). For PS2 cybrid, clones carrying 60, 70 and 80% mutant DNA were analysed (Fig. 2C). Clones with the same percentage in both lines were not obtained due to the oscillations suffered during cell culture. PS2 cybrid clones with a percentage below 60% were impossible to maintain.

**Fig. 5.**
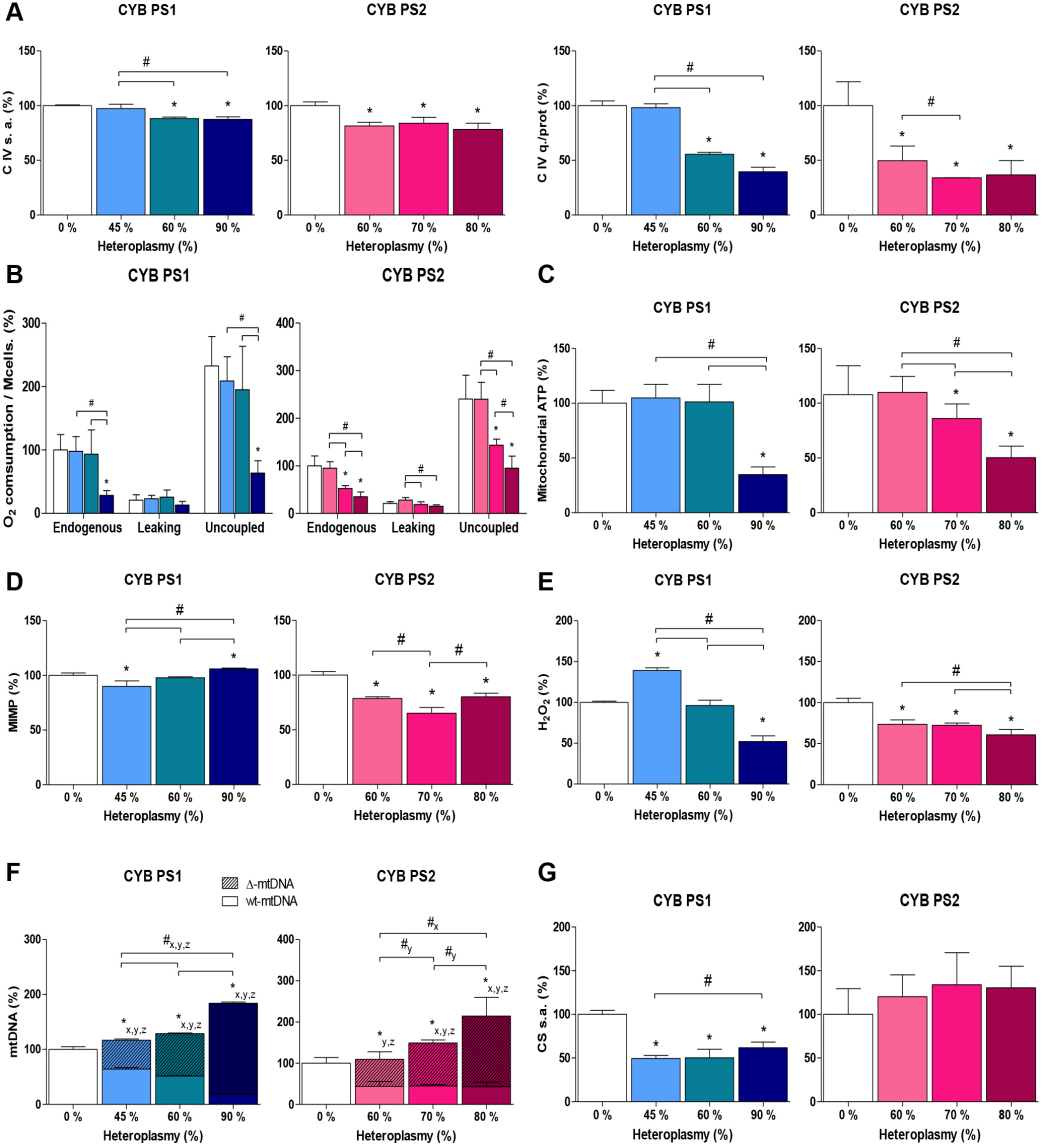
**Determination of bioenergetic pathologic threshold levels in cybrids.** (A) Respiratory CIV specific activity (s.a.) and quantity (q.) normalised for total protein (*N*=3). (B) Oxygen consumption (*N*=3). (C) Mitochondrial ATP levels (CYB PS1, *N*=6; CYB PS2, *N*=11). (D) MIMP (*N*=3). (E) H_2_O_2_ production (*N*=3). (F) mtDNA copy number (*N*=3). (G) Citrate synthase (CS) specific activity (*N*=3). Bars indicate the mean value of each clone relative to that of control clone in percentage and s.d. of independent experiments. Statistically significant differences were determined by a Mann–Whitney test (*P*<0.05) and represented by * (versus control), # (versus mutant clones) and subscripts x (total mtDNA), y (Δ-mtDNA) and z (wt-mtDNA).

CIV quantity and activity were significantly reduced in the clones derived from Pearson syndrome patients carrying 60% deletion or more. This decrease was more severe in quantity than in complex activity. No CIV failure was seen in PS1 cybrid cells with a deletion below 50% (Fig. 5A). Therefore, it seems that with 50% of complete mtDNA molecules there is an amount of CIV comparable to a non-deletion-carrying cell.

When mitochondrial respiration was analysed by measuring the oxygen consumption, we observed a decrease in those cybrid clones carrying a percentage of deletion of ∼70% or higher, in terms of basal and uncoupled respiration. Leaking respiration, measured by the addition of oligomycin to inhibit ATP synthase, did not differ with respect to the cells without deletion for any of the cases studied. In those carrier lines with a 60% deletion, and therefore with a lower amount of CIV, the oxygen consumption was not altered (Fig. 5B).

The same trend was observed for mitochondrial ATP levels. There were no differences between control cells and cells carrying 45% and even 60% of mutant mtDNA. Only a significant reduction in the amount of ATP was found when the deletion load exceeded 60% (Fig. 5C). This decrease was severe in both cybrid lines when the percentage of mutation was similar to that found in the blood of patients (85%) and close to that in fibroblasts. However, this reduction was more drastic in the cybrid carrying the smallest deletion but exhibiting the highest heteroplasmy.

The mitochondrial inner membrane potential (MIMP) was found to be lower in cells with 6514-bp deletion (CYB PS2) compared to their non-deletion control (Fig. 5D). All three CYB PS2 deletion-carrying clones reduced their MIMP by 20%, so it seems that an increase in the number of damaged mtDNA molecules does not cause greater deterioration in the electrochemical gradient; the minimum MIMP for this cell line may have been reached. These results did not occur in the cybrid line with the common deletion (CYB PS1), which showed a drop in membrane potential with 45% of deletion but not with higher heteroplasmy levels (Fig. 5D). Moreover, it seems that not only the reduction of MIMP causes the decrease in the synthesis of mitochondrial ATP, as we only see defective ATP production from 70% of deletion in CYB PS2 even though the MIMP is similar to the cybrids with 60% of deleted molecules.

The production of reactive oxygen species (ROS), as a reflection of the malfunctioning damaged mitochondrial chain, was measured. We observed differences between the two cybrid lines again, although the tendency was the same: the highest percentage of deletion result in the lowest ROS (Fig. 5E).

The number of mtDNA molecules was increased in all deletion-bearing cybrids except for CYB PS2 with 60% of heteroplasmy, in which there was a tendency to increase but this was not significantly reflected by the variability of the data. mtDNA levels doubled in cybrids most affected by the deletion, as a result of an attempt by cells to compensate for mitochondrial damage. However, most new mtDNA copies were deleted molecules (Fig. 5F). Moreover, the activity of citrate synthase did not increase in any of the deletion-bearing cybrids but was reduced in cybrids carrying the common deletion (Fig. 5G). According to these results, the threshold level of heteroplasmy seemed to be above 60% for both cybrids in spite of the different size of their deletions.

### Mitochondrial ultrastructure is affected in all Pearson syndrome cell models

Electron microscopy was used to analyse the ultrastructural morphology of mitochondria in cell models carrying mtDNA deletions. Mitochondria modulate their shapes by fusion and fission processes to adapt to specific cellular demands. As these changes can be induced by physiological processes, such as nutrient stress, it is common to see different mitochondrial morphologies in a healthy cell (Giacomello et al., 2020). However, important deviations have been found in cells with mitochondrial diseases (Liu et al., 2012; Komulainen et al., 2015; Vincent et al., 2016; Felczak et al., 2017; Molnar and Kovacs, 2018).

**Fig. 6.**
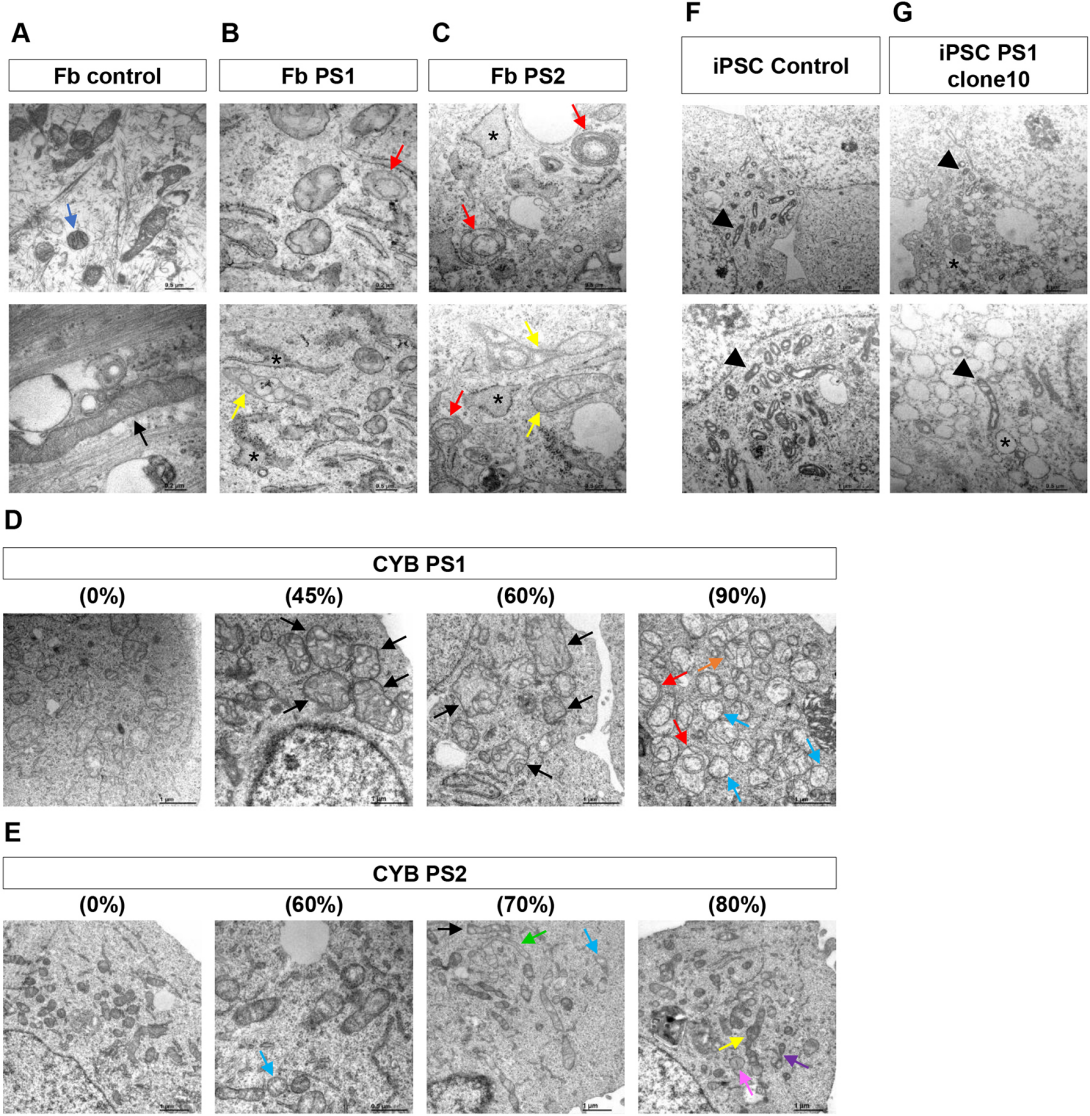
**Mitochondrial ultrastructure in different cell models carrying mtDNA deletions and controls.** (A) Control fibroblasts (Fb) showed elongated (black arrow) and spherical (blue arrow) mitochondria with a well-defined cristae network. (B,C) Electron micrographs of PS1 and PS2 fibroblasts showing swollen mitochondria with abnormal compartments bound by a double membrane and isolated from mitochondrial membrane (yellow arrows), and displaying concentric ‘onion-shaped’ cristae (red arrows). The cytoplasm also contained dilated rough endoplasmic reticulum with electron-dense ribosomes (asterisks). (D) CYB PS1 clones carrying 45% and 60% of deleted mtDNA showed giant rounded mitochondria with irregularly spaced cristae forming abnormal compartments (black arrows). The 90% deletion carrier clone presented rounded mitochondria with electron-light matrix and linearised cristae displaying geometrical shapes (orange arrow), concentric cristae forming ‘onion-like’ mitochondria (red arrows) or mitochondria with few or zero numbers of cristae (blue arrows). (E) CYB PS2 with 60% and 70% heteroplasmy presented normal tubular-shaped mitochondria (black arrow), mitochondria with an aberrant and poor system of cristae (blue arrows), and some branched mitochondria (green arrow). The CYB PS2 clone carrying 80% deletion showed many mitochondria with abnormal compartments with different electron density (yellow arrow), paracrystalline inclusions (pink arrow) and constricted mitochondria with nanotunnels formed by inner and outer mitochondrial membrane (purple arrow). (F,G) Electron micrographs of iPSC control and PS1 clone 10 showing round and tubular-shaped perinuclear mitochondria with underdeveloped cristae, electron-light matrix and a thicker and electron-dense intermembrane space (black arrowheads). iPSC PS1 clone 10 also presented dilated rough endoplasmic reticulum (asterisks). Scale bars: 1 µm (D; E, 0%, 70% and 80%; F; G, top); 0.5 µm (A, top; B,G, bottom; C; E, 60%); and 0.2 µm (A, bottom; B, top).

Normal mitochondria ultrastructure, defined by typical tubular cristae, crista junctions and electron-dense matrix, was mostly observed in control fibroblast. They presented elongated and spherical mitochondria with a well-defined cristae network (Fig. 6A). Interestingly, PS1 and PS2 fibroblasts exhibited more rounded and swollen mitochondria with few and damaged cristae, and an electron-light matrix. Furthermore, abnormal compartments bound by a double membrane and completely isolated from the mitochondrial membrane were frequently observed. In many cases, these mitochondria also displayed concentric ‘onion-shaped’ cristae (Fig. 6B,C). Additionally, electron micrographs of both Pearson syndrome fibroblasts displayed some branched mitochondria and dilated rough endoplasmic reticulum with electron dense ribosomes (Fig. 6B,C), which were not observed in control fibroblasts.

Although alterations in shape and ultrastructure were found in PS1 and PS2 cybrids carrying different degrees of deleted mtDNA, these were different despite similar levels of heteroplasmy. PS1 cybrid clones carrying 45% and 60% of deleted mtDNA showed giant rounded mitochondria with irregularly spaced cristae forming abnormal compartments (Fig. 6D). We did not observe important variations in size in PS2 cybrids with 60% and 70% of heteroplasmy but most of their mitochondria presented an aberrant and poor system of cristae. Some branched mitochondria were also detected in these PS2 cybrid clones (Fig. 6E). Furthermore, aberrations were especially pronounced when heteroplasmy was highest. Ultrastructural analysis of PS1 and PS2 cybrid clones with the highest heteroplasmy level revealed numerous mitochondria with few or no cristae inside (Fig. 6D,E). PS1 cybrid with 90% of deleted mtDNA showed rounded mitochondria with electron-light matrix and disorganized cristae. Nevertheless, most mitochondria exhibited linearised cristae displaying geometrical shapes, or concentric cristae without any connection to inner mitochondrial membrane forming ‘onion-like’ mitochondria (Fig. 6D). The PS2 cybrid clone carrying 80% of deletion showed many mitochondria with abnormal compartments, and in many cases with different electron density. This clone also exhibited paracrystalline inclusions inside some mitochondrial matrices (Fig. 6E). Regarding the shape, constricted mitochondria and nanotunnels formed by inner and outer mitochondrial membrane were observed (Fig. 6E).

Mitochondrial ultrastructure in iPSCs carrying the common mtDNA deletion (PS1 clone 10), and in a control line, was also analysed (Fig. 6F,G). Both cell lines mainly exhibited round-shaped perinuclear mitochondria with underdeveloped cristae and electron-light matrix. Nonetheless, some tubular mitochondria were also observed. In PS1 iPSC mitochondria, the cristae structure was poorer, or even completely missing (Fig. 6G). Electron micrographs also revealed that control and PS1 iPSCs seemed to have mitochondria with a thicker and electron-dense intermembrane space (Fig. 6F,G). Additionally, PS1 iPSC mitochondria showed abnormal compartments with different electron densities and dilated rough endoplasmic reticulum, similar to the rest of the PS1 models described previously (Fig. 6G).

According to these results, electron micrographs revealed abnormal architecture and morphology of mitochondria in all deletion carrier lines. We also observed that some alterations in shape, size and cristae structure were different depending on the Pearson syndrome cell model, which may be due to their different energy demands.

## DISCUSSION

Pearson syndrome is still a challenge in medicine as its evolution is usually tragic, leading to death in childhood. Patients who survive eventually develop Kearns–Sayre syndrome (Farruggia et al., 2018). Therefore, the development of suitable models for the study of new therapeutic approaches is necessary. Here, we report three cell models for the study of Pearson syndrome harbouring defective mtDNA molecules. In all cases, we observed the consequences of deleted mtDNA molecules, as mutant cells showed a drop in the amount of mitochondrial ATP, activity and amount of CIV and basal oxygen consumption. Furthermore, they all exhibited an increase in mtDNA copy number in an attempt to compensate for mitochondrial dysfunction. Interestingly, mitochondrial mass was not increased in cybrids or iPSCs, unlike the primary culture of fibroblasts, which showed increased citrate synthase activity. Alterations at the ultrastructural level were also observed in the mitochondria, reflecting some features usually linked to the presence of mtDNA mutations. Clones of the cybrids with different deletion levels allowed us to establish the threshold heteroplasmy percentage at 60-70%, from which deleterious effects of the deletion were revealed.

Our study allowed us to compare the influence of the deletion on three different cell models, confirming their usefulness for demonstrating the pathogenicity of the deletions and giving value to each one depending on the availability of tissue or the experiment to be carried out. However, they all have advantages and also limitations. Primary culture with fibroblasts allows us to work directly with patient samples with minor manipulation but with proliferative capacity for a few passages. In this cell model, the patient's nuclear and mitochondrial backgrounds are normally conserved; therefore, some effects, such as compensation mechanisms, could mask the direct consequences of the deletion and hinder the comparative analysis with controls. Mitochondrial biogenesis is considered a compensatory mechanism as it is observed in skeletal muscle cells of patients with mtDNA-related diseases (DiMauro and Schon, 2003). According to this, our deletion-bearing fibroblasts exhibited an increase in mitochondrial mass estimated by the specific activity of citrate synthase. Nevertheless, reports related to mtDNA deletion in fibroblasts fundamentally study the evolution of heteroplasmy level during culture and/or the distribution of deleted mtDNA at intercellular and intracellular levels (Bourgeron et al., 1993; Van De Corput et al., 1997; Van Den Ouweland et al., 2000). It has been reported that fibroblasts experience a rapid loss of the deleted mtDNA during culture (Van Den Ouweland et al., 2000), even when maintaining the cells with uridine, which has been shown to help the growth of those cells with respiratory chain defects (Bourgeron et al., 1993; Gérard et al., 1993). Our primary fibroblast cultures also showed a slight variation in mutation load across passages but kept heteroplasmy levels greater than 80%.

Cybrids have been the most used model for studying the pathogenicity of mtDNA deletions because they allow the homogenization of the nuclear genetic background by fusion of the platelets or enucleated fibroblasts of patients and controls with the same nuclear background (Hayashi et al., 1991; Porteous et al., 1998; Van Den Ouweland et al., 2000; Diaz, 2002; Pallotti et al., 2004; Jou et al., 2005; Comte et al., 2013). However, they present an aneuploid nucleus with a large number of chromosomes, which is very different from a normal cell. Our cybrids showed a tendency to either lose the mutant DNA or to increase levels above 50%. It has been proposed that deleted mtDNA would be lost when the level of heteroplasmy was low, based on the advantage in growth of cells without deletion (Moraes et al., 1989; Hayashi et al., 1991). However, when the mutation load was high, there would be a tendency to increase due to the replicative advantage of smaller DNA molecules (Hayashi et al., 1991). Nevertheless, other authors working with Pearson syndrome cybrids in a different nuclear background did not find remarkable variation in heteroplasmy (Van Den Ouweland et al., 2000). In this sense, the nuclear genetic background of the recipient ρ^0^ cell could influence the segregation of mutant and wild-type mtDNA in cybrids with mitochondrial mutations (Dunbar et al., 1995).

iPSCs bearing an mtDNA deletion, the most wanted model in recent years as it allows the study of specific affected tissue, have been described in only a few cases (Cherry et al., 2013; Russell et al., 2018; Peron et al., 2021). iPSCs conserved the patient's genetic background and they have the advantage of being euploid cells. However, these iPSC models are difficult to generate. In our study, the generation of deletion-bearing iPSCs was only possible for the PS1 line. To achieve this, three independent reprogramming experiments were necessary. The change of the culture conditions to NutriStem medium would have facilitated the metabolic shift towards glycolysis, minimizing the impact of high heteroplasmy on the iPSCs, as noted previously (Russell et al., 2018). Different tendencies of heteroplasmy have been reported in deletion-bearing iPSCs (Cherry et al., 2013; Russell et al., 2018). The amount of deleted mtDNA remained at 75% in our Pearson syndrome iPSC lines for at least 20 passages; however, we observed some fluctuations when cultures were maintained over 30 passages. Growth in high glucose with uridine, and the avoidance of prolonged cell culture, would probably help to maintain mutant cells.

Specific energy requirements of each cell type may be the reason of some differences observed among Pearson syndrome cell models. Tumour cells and iPSCs are more dependent on glycolysis; therefore, secondary effects of mitochondrial mutation, such as mitochondrial biogenesis, could not come about. Additionally, it has been shown that a decrease in biogenesis and mitochondrial function seems to be essential for the reprogramming and maintenance of iPSCs (Hsu et al., 2016). The minor dependency of iPSCs on mitochondrial function has also been revealed in their immature-like mitochondria ultrastructure without well-developed cristae, which has been previously described as a common feature of stem cells (Prigione et al., 2010; Folmes et al., 2012). Otherwise, an increase in the content of mtDNA was evident in all our deletion-bearing cells, regardless of their metabolic profile, and especially in cells derived from the patient with the largest deletion (PS2). However, the increase in copy number is often not related to an increase in wild-type mtDNA genome (Stewart and Chinnery, 2015). In fact, in the specific case of deletions, an increase in mtDNA levels is usually accompanied by an increase in the percentage of deleted copies as they have a replicative advantage (Wallace, 1992; Diaz, 2002; Russell et al., 2018), which is a trend reflected in our cybrid lines.

Despite the cell models presented in this work reflecting the mitochondrial failure produced by the presence of mtDNA deletions, other specific parameters did not vary as expected. MIMP is generated by the respiratory chain being coupled to electron transport, and is used to synthesise ATP (James et al., 1996; Porteous et al., 1998). Surprisingly, our mutant cybrids did not always show a drop in the MIMP, and when they did it was not proportionate with the percentage of deletion. Nevertheless, the amount of mitochondrial ATP decreased as the number of deleted molecules increased. Therefore, other factors, such as the involvement of subunits of the ATP synthase complex in the deletion, could be decisive in this result. ROS production has also proposed to be a mechanism associated with respiratory chain dysfunction and partly responsible for the cell deterioration observed in mitochondrial pathologies (Esposito et al., 1999; Peng et al., 2006). Despite an increase in ROS being associated with deletion-bearing cybrids (Jou et al., 2005; Peng et al., 2006; Indo et al., 2007), our cybrid lines did not show an increase in ROS when high levels of deletion were observed, and even exhibited a decrease compared to controls without deletion. The presence of fewer complete transport chains may be one of the causes. In fact, it has been shown that the production of ROS is significantly inhibited when mtDNA is completely eliminated (ρ^0^) (Peng et al., 2006).

The relationship between some features of mtDNA deletions and the severity of the disease has been controversial. Published studies have been carried out at the tissue level, in skeletal muscle, revealing an association among heteroplasmy, size or location of deletions with the clinical phenotype (López-Gallardo et al., 2009) and disease progression (López-Gallardo et al., 2009; Grady et al., 2014). However, other authors consider that the mutation load is not predictive of either onset or phenotype, or quite the contrary (Yamashita et al., 2008; Sadikovic et al., 2010; Jeppesen et al., 2020). In our *in vitro* models, we were able to observe that the size and load of the deletion influenced the severity of the phenotype, depending on the parameter analysed or the cell type studied. Some parameters of mitochondrial function, such as CIV activity, were more affected in those lines carrying the largest deletion, regardless of the mutation load. This result was expected as the deletion in PS2 involves a greater number of CIV subunit genes. However, when we analysed the global function of the electron transport chain by measuring mitochondrial ATP production and basal oxygen consumption, we observed that heteroplasmy level appeared to have a greater influence than the number of deleted mtDNA genes.

Hence, the balance between mutated and wild-type mtDNA is essential as it will define the number of complete electron transport chains and will be decisive in the expression of the pathological phenotype. This threshold level depends on the mtDNA mutation and the cell type affected (Durham et al., 2007), but it has been reported to be usually greater than 80%, suggesting that most mtDNA mutations are haploinsufficient or recessive (Durham et al., 2007; Stewart and Chinnery, 2015). In our deletion-bearing cybrids, the threshold level was established above 60%, as no global biochemical defects were found when the heteroplasmy was at this level or lower. Other authors have also suggested the threshold is 75%, when carrying out mitochondrial translation studies in Pearson syndrome cybrids (Van Den Ouweland et al., 2000). Curiously, successive analysis of deletion load in blood samples of PS1 showed a heteroplasmy reduction below the pathologic threshold level established in cells, by the time the patient became non-transfusion dependent. Regarding this event, some authors have suggested that mutant mtDNA genomes tend to be lost in blood, whereas they accumulate in postmitotic tissues, such as muscle (Van Den Ameele et al., 2020). In any case, reversing the deleterious effect caused by the deletions would be the only hope for curing these patients. Our results suggest that strategies to reach 40% of complete mtDNA molecules would be effective in rescuing mitochondrial function in affected tissues. Current therapies for Pearson syndrome are mainly focused on palliating symptoms through cocktails of nutritional supplements and vitamins, achieving a moderate but not definitive effect (Gorman et al., 2016). Treatment with antioxidants, such as co-enzyme Q10 or vitamin C and E, is common for patients with mitochondrial pathologies, although their clinical efficacy has been questioned (Garone and Viscomi, 2018). Mitochondrial mass modulation is another possible treatment strategy, either by favouring mitochondrial biogenesis (Viscomi et al., 2015; Filograna et al., 2019) or by regulating mitophagy (Bielas et al., 2018). However, achieving an increase mainly in complete mtDNA molecules would probably be more complicated in the case of deletions. A ketogenic diet, which leads the metabolism towards β-oxidation of fatty acids, has been demonstrated to be effective in reducing mutant mtDNA in cybrids carrying a single deletion (Santra et al., 2004) and a Leber hereditary optic neuropathy point mutation (Emperador et al., 2019). Cellular models developed and well characterised in this work could be valuable for testing these treatments, by providing relevant and necessary information for the transfer to clinical practice.

Interestingly, electron micrographs of all deletion-bearing cells revealed important deviations from normal mitochondrial ultrastructure. As the mitochondrial morphology constantly varies with metabolic requirements (Giacomello et al., 2020), any damage in OXPHOS will probably trigger important structural changes. Most of the ultrastructure alterations observed in our models have been previously associated with different mitochondrial pathologies (Liu et al., 2012; Komulainen et al., 2015; Felczak et al., 2017). Our Pearson syndrome lines showed swollen mitochondria with electron-clear matrices and disorganised cristae that were sometimes concentric shaped. These structures have been previously observed in skeletal muscle biopsies and small blood vessels from mitochondrial encephalopathy, lactic acidosis and stroke-like episodes (MELAS) syndrome patients (Felczak et al., 2017). Aberrant compartments mainly observed in Pearson syndrome cybrids have been reported in previous studies in a patient with a m.8344A>G mutation and in a chronic progressive external ophthalmoplegia (CPEO) patient (Vincent et al., 2016). Additionally, this author has described non-identical ultrastructural abnormalities among skeletal muscle biopsies from patients with mtDNA deletions (Vincent et al., 2016). Therefore, it does not seem possible to determine a type of ultrastructural alteration especially associated with mtDNA deletions. Rather, alterations can only be associated with all mitochondrial pathologies.

In conclusion, as there are still many unknowns about Pearson syndrome, the generation of a great number of different cell models may be essential for understanding the disease and for studying new treatments. As we have shown, all deletion-bearing cell models were seriously affected by the mtDNA deletion, regardless of their different bioenergetic requirements. Additionally, some aspects, such as the transition to Kearns–Sayre syndrome or tissue-specific manifestations like pancreatic or hematopoietic failure, might be investigated using an experimental model that allows differentiation to specific lineages. Therefore, despite all of the cell models presented here providing relevant information about the pathogenesis of the disease, we consider that the use of iPSCs is essential for advancing knowledge about this syndrome and for the development of more efficient and personalised therapies. Further studies in this field will be necessary to produce a significant impact on patients.

## MATERIALS AND METHODS

### Pearson syndrome cell models: fibroblasts, cybrids and iPSCs. Cell culture conditions, reprogramming and differentiation

To homogenise nuclear and environmental factors, we produced transmitochondrial cell lines (cybrids) with the osteosarcoma 143B rho^0^ nuclear background using patient cytoplasts. For this, cytoplasts were generated from patient fibroblasts and were fused with osteosarcoma 143B rho^0^ cells (Bayona-Bafaluy et al., 2003). These cybrids, as well as primary fibroblasts from Pearson syndrome patient skin explants and control cells, were maintained in Dulbecco's modified Eagle medium (DMEM; Thermo Fisher Scientific) with no antibiotics and containing glucose (4.5 g/l), pyruvate (0.11 g/l), uridine (50 µg/ml) and 5% or 10% of fetal bovine serum (FBS). For cellular and biochemical studies, fibroblasts and cybrids cells were cultured for 72 h in low glucose (5 mM) DMEM supplemented with uridine and FBS with no antibiotics.

Fibroblasts were reprogrammed into iPSCs using a Cytotune-iPS 2.0 Sendai Reprogramming kit (Thermo Fisher Scientific) following the manufacturer's instructions. Nascent iPSCs were cultured for 3-4 weeks after transduction on feeder layers in iPSC medium containing KnockOut DMEM/F-12, 20% of Knockout Serum Replacement, 1× MEM non-essential amino acids solution, 1× GlutaMAX-I, β-mercaptoethanol (100 µM), 1× penicillin/streptomycin and bFGF (4 ng/ml; Thermo Fisher Scientific), supplemented with pyruvate (0.11 g/l) and uridine (50 µg/ml). Then, emerging iPSC colonies were mechanically picked and expanded, both on feeder layers with iPSC medium and on vitronectin-coated plates with Essential 8 culture medium (Thermo Fisher Scientific) supplemented with uridine and pyruvate, until no Sendai virus genome was detected (Fig. S2) and lines were stable. Subsequently, iPSCs were maintained on vitronectin-coated plates with NutriStem hPSC XF medium (Biological Industries) supplemented with uridine and pyruvate. Cells were passaged every 3-4 days using 0.5 mM EDTA, and medium was replaced daily. *In vitro* differentiation of iPSCs into the three germ layers was performed by adapting a protocol described previously (Galera-Monge et al., 2016). No bacteria, fungi, yeast or mycoplasma contamination was detected in the different cell models.

All samples were collected with written informed consent, and the Ethics Review Committees of the involved hospitals and the Government of Aragón approved the study (Comité Ético de Investigación Clínica de Aragón-CEICA-12/2014).

### Genetic analysis

Total DNA was extracted from the different biological samples by conventional methods. mtDNA deletions were detected and analysed by long-range PCR using the following primers: forward, 5′-ACCGCCCGTCACCCTCCTCAAGTATACTTCAAAGG-3′; and reverse, 5′-ACCGCAGGTCCTTTGAGTTTTAAGCTGTGGCTCG-3′. Heteroplasmy was determined by Southern blotting, as described previously (Ascaso et al., 2010), or qPCR using a StepOne Real-Time PCR System (Applied Biosystems) and specific Taqman probes (Thermo Fisher Scientific) for *MT-RNR1* and *MT-ND4*, respectively located outside and inside deleted region. The Ct comparative method to a non-deletion bearing sample was used for heteroplasmy calculations. mtDNA copy number was also quantified by qPCR using a StepOne Real-Time PCR System as described previously (Andreu et al., 2009). Three independently isolated samples were measured in triplicate for mtDNA content and heteroplasmy determinations.

Cytogenic analyses were performed by Citogen (Center of Genetic Analysis, Zaragoza). At least 21 metaphase cells were analysed for each cell type. The genetic fingerprint of cell models was determined using an AmpFLSTR Identifiler PCR Amplification Kit (Thermo Fisher Scientific) and an ABI Prism 3730xl DNA analyser (Applied Biosystems) at the Unidad de Genomica, Universidad Complutense de Madrid. These genetic fingerprints were compared to the American Type Culture Collection cell lines or their respective original line. The complete mtDNA was amplified and sequenced according to protocols described previously (Gómez-Durán et al., 2010). The revised Cambridge reference sequence (GenBank, NC_012920) and an mtDNA phylogenetic tree were used to locate mutations and define mtDNA haplogroups (Van Oven and Kayser, 2009).

Analysis of the expression of exogenous reprogramming vectors was performed by RT-PCR following the manufacturer's instructions (Cytotune-iPS 2.0 Sendai Reprogramming Kit, Thermo Fisher Scientific). Total RNA was isolated using a NucleoSpin RNA II kit (Macherey-Nagel). Total RNA (1 µg) was reverse transcribed using a Transcriptor First Strand cDNA Synthesis Kit (Roche). Then, PCR was performed using Sendai virus-specific primers, defined in the manufacturer's instructions, and analysed by agarose gel electrophoresis.

### Immunofluorescence

For fluorescent microscopy, the cultured cells were fixed with 4% paraformaldehyde for 15 min at room temperature and permeabilised with 0.1% Triton X-100 for 10 min. After blocking for 30 min with 5% bovine serum albumin, the washed cells were incubated for 1 h at room temperature or overnight at 4°C with a primary antibody against the POU domain, class 5, transcription factor 1 (OCT4) (1:200, ab19857, Abcam), Transcription factor SOX-2 (SOX2) (1:200, ab92494, Abcam), Homeobox protein NANOG (NANOG) (1:100, ab109250, Abcam), Tra-1-60 (1:150, MAB4360, Merck), Tubulin β-3 (TUBB3) (1:500, ab18207, Abcam), smooth muscle alpha actin (SMA) (1:400, A2547, Merck) or α-fetoprotein (AFP) (1:200, ab3980, Abcam). Subsequently, the cells were incubated with fluorescence-labelled secondary Alexa Fluor 594 or 488 (1:1000, A11001, A11008 and A11012, Invitrogen) at room temperature for 1 h, protected from light. The cells were further incubated with 1 µM DAPI for nuclear staining. Between incubations, samples were washed with PBS containing 0.1% Triton X-100. Alkaline Phosphatase Live Stain 500X from Thermo Fisher Scientific was used for determining the enzyme activity following the manufacturer's instructions. Pictures were acquired using a Floyd Cell imaging station (Life Technologies).

### Biochemical analysis

Determination of CIV activity and quantity was performed using Mitoprofile Human CIV Activity and Quantity (Mitosciences, Abcam) according to a protocol described previously (Emperador et al., 2015).

Mitochondrial ATP amount, normalised by the cell number, was measured using the CellTiter-Glow Luminiscent Cell Viability Assay following previously described protocols with minor modifications (Emperador et al., 2019). Briefly, 20,000 cells/well for fibroblast analysis and 10,000 cells/well for cybrid analysis were seeded 14-16 h before measurement. Then, cells were washed twice with PBS and incubated for 2 h in record solution with 5 mM 2-deoxy-d-glucose plus 1 mM pyruvate (oxidative ATP production). For iPSCs, this incubation was performed in 100 mm diameter plates at 80% of confluency, and then, cells were collected and counted for measuring the ATP production of 20,000 cells/well. Cells were lysed, and lysates were incubated with luciferin/luciferase reagents. Samples were measured using a microplate luminometer, and the results referred to cell number.

Oxygen consumption was analysed using the high-resolution oxygraph OROBOROS (Oroboros Instrument, Innsbruck, Austria). Cells were collected, washed, counted and resuspended at 10^6^ cells/ml in DMEM. To correct for oxygen consumption not due to the electron transport chain, inhibition of mitochondrial respiration by potassium cyanide was performed. Each condition was analysed three times. Respiration was measured at 37°C, with chamber volumes set at 2 ml. The software DatLab (Oroboros Instrument, Innsbruck, Austria) was used for data acquisition at 1 s time intervals, as well as for data analysis (Gnaiger et al., 1995).

MIMP and H_2_O_2_ production quantifications were performed as described previously (Gómez-Durán et al., 2010; Llobet et al., 2015), with minor modifications. Citrate synthase was measured in 96-well plates, using freeze-thawing-treated total cell homogenate and a standard protocol (Kirby et al., 2007). Activity data were normalised for total protein. Microplate assays were performed in a NovoStar MBG Labtech microplate instrument, and a Beckman Coulter Cytomics FC500 cytometer was used for the measurement of intracellular fluorescence. Weasel software was used for flow cytometry data analysis.

All enzyme determinations were performed in triplicate in at least three independent experiments.

### Electron microscopy

Ultrastructural analysis was performed following a procedure described previously (Llobet et al., 2015). Briefly, cells were seeded in Permanox chamberslides (Nunc), fixed with 2.5% glutaraldehyde for 2 h at 4°C and maintained in phosphate buffer supplemented with 0.05% sodium azide. Postfixation was carried out using 1% OsO_4_ for 2 h. The cells were dehydrated in a graded series of ethanols up to absolute. The specimens were then passed through different mixtures of ethanol and araldite (3:1, 1:1 and 1:3), and embedded in pure araldite. After 3 days of polymerization at 70°C, ultrathin sections were cut and stained following a protocol published previously (Reynolds, 1963). The sections were examined with a JEOL 1010 transmission electron microscope using a Gatan Bioscan camera and Digital Micrograph software.

### Statistical analysis

The statistical package StatView 5.0 was used to perform all statistical analyses. Data are expressed as mean±s.d. The non-parametric Mann–Whitney test was used to evaluate the statistical significance between experimental groups. The number of sides was automatically selected by the statistical software. *P*<0.05 was considered statistically significant. All samples were measured at least in biological triplicates.

## Supplementary Material

Supplementary information
