# Supplementary material for: Development and characterization of cell models harbouring mtDNA deletions for in vitro study of Pearson syndrome
Source: Dis Model Mech. 2022 Mar 1;15(3):dmm049083. doi: 10.1242/dmm.049083 (PMC8906170; doi:10.1242/dmm.049083)
Supplement: Supplementary information [file dmm-15-049083-s1.pdf]

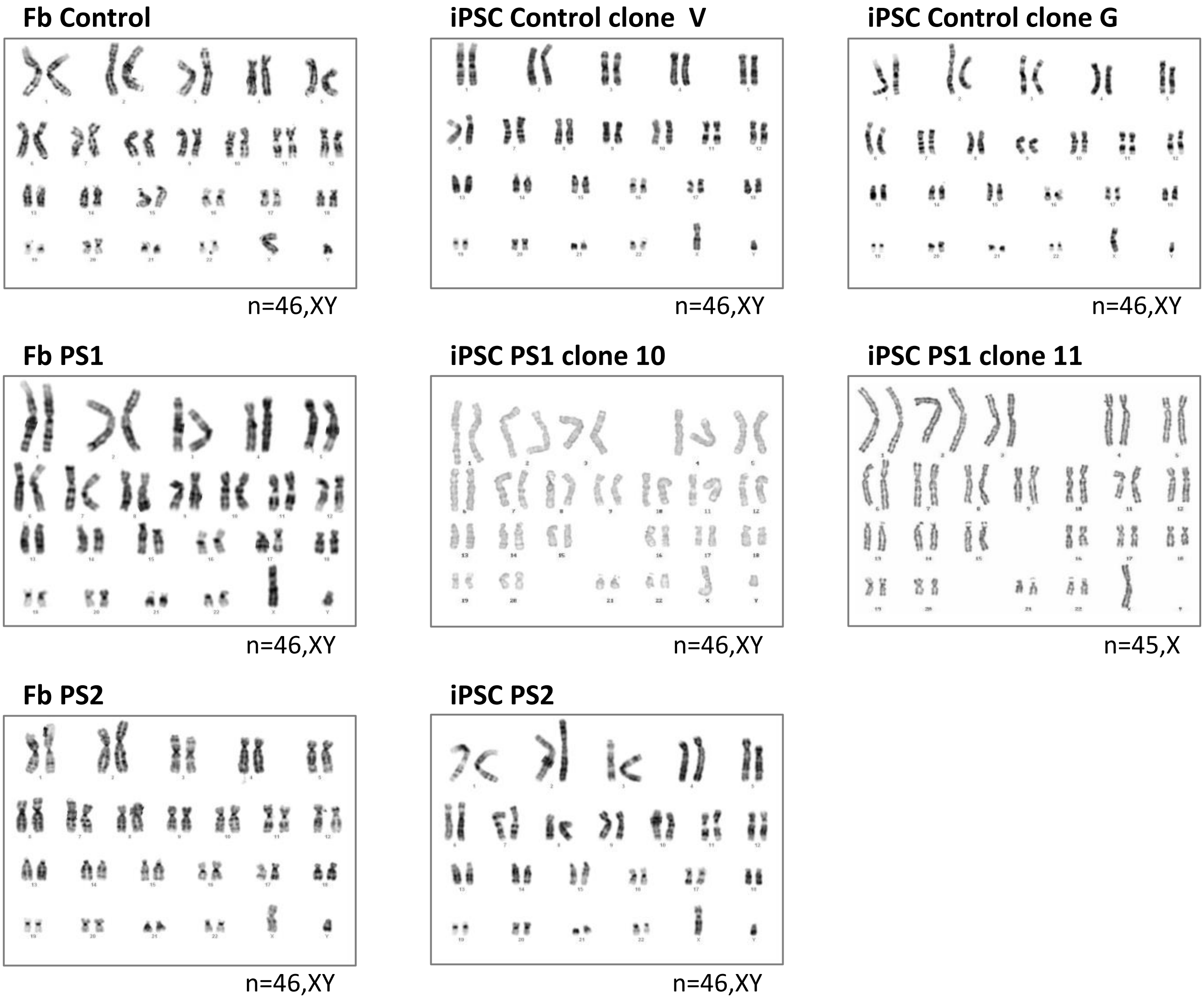

Fig. S1. Karyotype analysis of control and PS fibroblasts and iPSC. At least 21 metaphases were analyzed. All lines showed a normal karyotype (46, XY), but iPSC PS1 clone 11 which presented a monosomy in sex chromosomes (45, X).

Disease Models & Mechanisms • Supplementary information

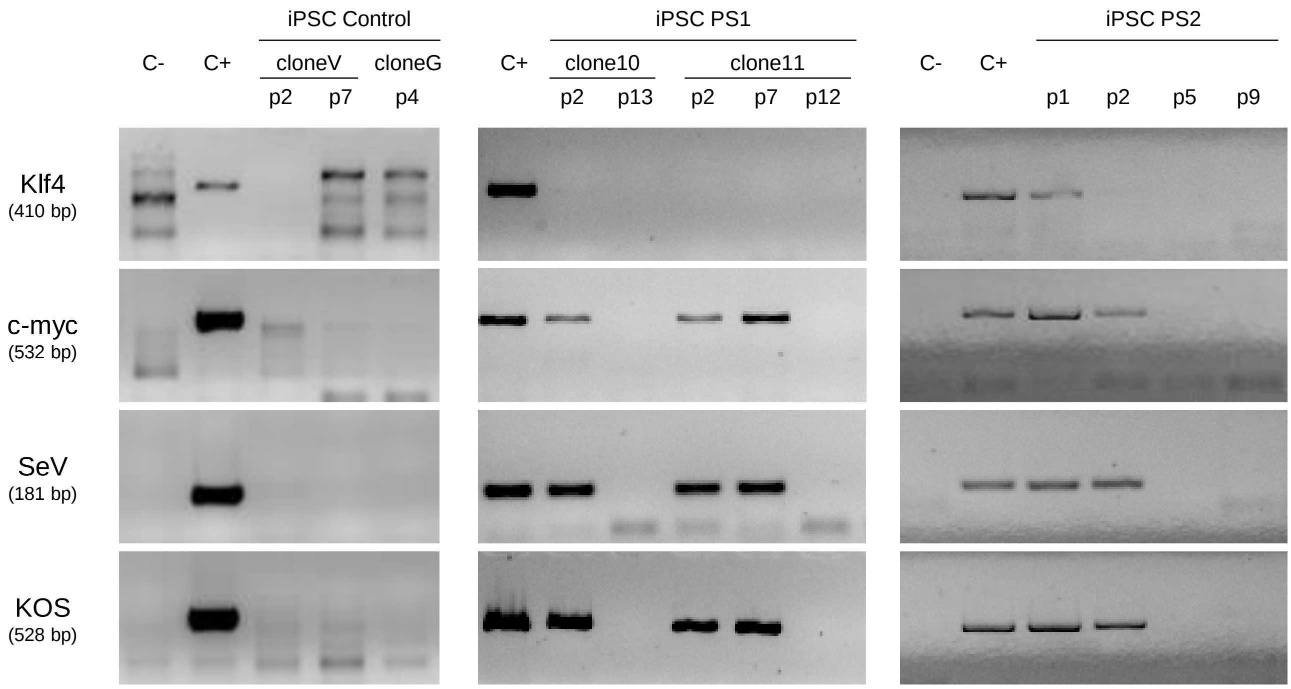

Fig. S2. Analysis of expression of exogenous reprogramming vectors in iPSC after some passages. Reprogramming vectors were Kruppel Like Factor 4 (*Klf4*), oncogene c-myc (*c-myc*) and KOS formed by factors *Klf4*, *Oct3/4* and *Sox2*. SeV genome was also detected (SeV). p indicates passage. Fibroblasts were used as negative controls (C-). Fibroblasts just after transduction were used as positive controls (C+).

Disease Models & Mechanisms • Supplementary information

Table S1. DNA fingerprint analysis of control and PS fibroblasts, cybrids and iPSC

	Alleles									
**Marker**	**Fb Control**	**iPSC Control clone V**	**iPSC Control clone G**	**Fb PS1**	**iPSC PS1 clone 10**	**iPSC PS1 clone 11**	**Fb PS2**	**143B**	**CYB PS1**	**CYB PS2**
**TPOX**	8,9	8,9	8,9	8	8	8	11	11	11	11
**D2S1338**	17,20	17,20	17,20	19	19	19	19		24,25	24,25
**D3S1358**	18	18	18	15	15	15	15,16		15	15
**FGA**	24,25	24,25	24,25	22	22	22	22,25		24	24
**D5S818**	11	11	11	10,12	10,12	10,12	11,12	13	13	13
**CSF1PO**	11,12	11,12	11,12	11	11	11	10,12	12	12	12
**D7S820**	9,10	9,10	9,10	9	9	9	11,12	11,12	11,12	11,12
**D8S1179**	13,14	13,14	13,14	13,15	13,15	13,15	14,15		11,14	11,14
**TH01**	7,9	7,9	7,9	8.3,9	8.3,9	8.3,9	6,1	6	6	6
**vWA**	15,17	15,17	15,17	16,17	16,17	16,17	16,18	18	18	18
**D13S317**	10,12	10,12	10,12	8,12	8,12	8,12	8,12	12	12	12
**D16S539**	11,13	11,13	11,13	12,13	12,13	12,13	11,12	10,13	10,13	10,13
**D18S51**	16,19	16,19	16,19	15	15	15	12		17	17
**D19S433**	12,15.2	12,15.2	15.2	14	14	14	13,14		13	13
**D21S11**	29	29	29	32.2	32.2	32.2	27,32		31.2, 32.2	31.2, 32.2
**AMEL**	X,Y	X,Y	X,Y	X,Y	X,Y	X	X,Y	X	X	X

Disease Models & Mechanisms • Supplementary information
